# Supplementary material for: Nav1.8-expressing neurons control daily oscillations of food intake, body weight and gut microbiota in mice
Source: Commun Biol. 2024 Feb 22;7:219. doi: 10.1038/s42003-024-05905-3 (PMC10883928; doi:10.1038/s42003-024-05905-3)
Supplement: Supplementary file 2 — Supplemental Information [file 42003_2024_5905_MOESM2_ESM.pdf]

Supplementary Tables

Supplementary Table 1: Primer pairs and sequences used to detect Nav1.8 knock-in *Cre* and floxed *DTA* alleles and their respective wild-type fragments.

<table>
<tr><th>Genotype</th><th colspan="2">Primer pairs</th><th>Name of primer´s pairs</th><th>Amplicon length</th></tr>
<tr><td rowspan="3">Wild-type or Nav1.8 knock-in Cre-recombinase</td><td colspan="2">Common forward / wild-type reverse</td><td>mSNS13s/mSNS12a</td><td>438bp</td></tr>
<tr><td colspan="2">Common forward / Nav1.8 knock-in Cre reverse</td><td>mSNS13s/Cre5a</td><td>461bp</td></tr>
<tr><td colspan="2">Nav1.8 knock-in Cre forward / Nav1.8 knock-in Cre reverse</td><td>Cre2s/Cre5a</td><td>249bp</td></tr>
<tr><td rowspan="2">Wild type or floxed DTA</td><td colspan="2">Common forward / DTA reverse</td><td>ROSA26 F/floxed DTA</td><td>340bp</td></tr>
<tr><td colspan="2">Common forward / common reverse</td><td>ROSA26 F/ROSA 26 R</td><td>650bp</td></tr>
<tr><th>Primer name</th><th colspan="4">5´-3´sequence</th></tr>
<tr><td>mSNS13s</td><td colspan="4">TGT AGA TGG ACT GCA GAG GAT GGA</td></tr>
<tr><td>mSNS12a</td><td colspan="4">TTA CCC GGT GTG TGC TGT AGA AAG</td></tr>
<tr><td>Cre5a</td><td colspan="4">AAA TGT TGC TGG ATA GTT TTT ACT GCC</td></tr>
<tr><td>Cre2s</td><td colspan="4">CTG CAT TAC CGG TCG ATG CAA CGA</td></tr>
<tr><td>ROSA26 F</td><td colspan="4">AAA GTC GCT CTG AGT TGT TAT</td></tr>
<tr><td>floxed DTA</td><td colspan="4">GCG AAG AGT TTG TCC TCA ACC</td></tr>
<tr><td>ROSA 26 R</td><td colspan="4">GGA GCG GGA GAA ATG GAT ATG</td></tr>
<tr><th colspan="5">TaqMan® Gene Expression Assays used for validation of the Nav1.8 genetic ablations</th></tr>
<tr><td>Target name</td><td>Assay reference</td><td colspan="2">Assay ID</td><td>Amplicon length</td></tr>
<tr><td>Scn10a (Nav1.8)</td><td>4448892</td><td colspan="2">Mm00501467_m1</td><td>82bp</td></tr>
<tr><td>Rpl19</td><td>4331182</td><td colspan="2">Mm02601633_g1</td><td>69bp</td></tr>
</table>

Supplementary Table 2: Anti-mouse antibodies against immune markers to quantify immune cells in the spleen and intestinal epithelium and lamina propria by flow cytometry.

Immune cells	markers	antibodies	Supplier	References
**ILC1 group**	ILC1: Lin- Tbet+ IFNɣ+ CD49a+	PerCP-Cy™5.5-conjugated anti-Lineage antibody cocktail (LIN)	BD Bioscience	561317
	NK: Lin- Tbet+ IFNɣ+ CD49a-	PE-Vio615-conjugated anti-Tbet*	Miltenyi Biotec	130-107-611
		APC-conjugated anti-IFNɣ*	Miltenyi Biotec	130-109-723
		PE-vio 770- conjugated anti-CD49a	Miltenyi Biotec	130-107-590
**ILC2**	Lin- GATA3+ IL4+	PerCP-Cy™5.5-conjugated anti-Lineage antibody cocktail (LIN)	BD Bioscience	561317
		BUV395- conjugated anti-GATA3*	BD Bioscience	565448
		Pe-Cy7- conjugated anti-IL4*	Biolegend	504118
**ILC3**	Lin- RORɣt+ IL22+	PerCP-Cy™5.5-conjugated anti-Lineage antibody cocktail (LIN)	BD Bioscience	561317
		BV421- conjugated anti-RORɣt*	BD Bioscience	562894
		PE- conjugated anti-IL22*	Biolegend	516404
**Macrophages**	M1: F4/80+ CD80+ iNOS+	FITC-conjugated anti-F4/80	Miltenyi Biotec	130-102-327
	M2: F4/80+ CD206+ Arg1+	Pe-Vio770-conjugated anti-CD80	Miltenyi Biotec	130-102-372
		APC-conjugated anti-iNOS*	Thermo Fisher Scientific	17-5920-80
		PerCPCy5.5-conjugated anti-CD206	Biolegend	141716
		PE-conjugated anti-Arg1*	R&D Systems	IC5868P
**CD4+ T cells**	Treg: CD4+ CD25+ Foxp3+	BV711-conjugated anti-CD4	BD-Bioscience	563050
	Th17: CD4+ RORɣt+ IL17F+	APCCy7-conjugated anti-CD25	BD-Bioscience	557658
		PE-conjugated anti-Foxp3*	Miltenyi Biotec	130-111-678
		BV421- conjugated anti-RORɣt*	BD Bioscience	562894
		PerCP-Cy5.5 conjugated anti-IL17F*	BD Bioscience	562194
**CD4+ T cells**	Memory T cells: CD4+ CD44high CD62low	BV711-conjugated anti-CD4	BD Bioscience	563050
	Effector T cells: CD4+ CD44- CD62-	PerCP-Cy™5.5-conjugated anti-CD44	BD-Bioscience	560570
		APC-conjugated anti-CD62	BD-Bioscience	561919

antibodies against intracellular markers

Supplementary Table 3: Genes, primer pair sequences and conditions of the qPCR reactions

Gene name	Abbreviation	Sequence 5´- 3´	tissue
Neurogenic differentiation 1	***Neurod1***	Forward: AGGAATTCGCCCACGCAGAAG<br>Reverse: CTCCTCTGCATTCATGGCTTCAAG	duodenum, ileum and colon
Neurogenin 3	***Ngn3***	Forward: ACTCAGCAAACAGCGAAGAAG<br>Reverse: CAGTGCCCAGATGTAGTTGTG	duodenum, ileum and colon
Neuropeptide Y	***Npy***	Forward: AATCTCATCACCAGACAGAG<br>Reverse: CTTTCCTTCATTAAGAGGTCTG	hypothalamus
Melanocortin 4 receptor	***Mc4r***	Forward: GCGTTTCGAATGGGTCGGAAACCA<br>Reverse: CCGCAATGGAAAGCAGGCTGCAA	hypothalamus
Cholecystokinin A receptor	***Cck1r***	Forward: GACAGCCTTCTTATGAATGGGAG<br>Reverse: GCTGAGGTTGATCCAGGCAG	hypothalamus
Glucagon-like peptide 1 receptor	***Glp1r***	Forward: GGCGTCAACTTTCTTATCTTC<br>Reverse: CAAAGATGACTTCATGTGTCC	hypothalamus
Leptin receptor	***Lepr***	Forward: GGACACAGGTGGGACACTCT<br>Reverse: CCCCACAGCACATTTTTCTT	hypothalamus
Pro-opiomelanocortin-alpha	***Pomc***	Forward: AAAAGAGGTTAAGAGCAGTG<br>Reverse: ACATCTATGGAGGTCTGAAG	hypothalamus
Cocaine- and amphetamine-regulated transcript	***Cart***	Forward: AAGAAGTCCTGAAGAAGCTC<br>Reverse: CAAGCACTTCAAGAGGAAAG	hypothalamus
Agouti related neuropeptide	***Agrp***	Forward: AGGTCTAAGTCTGAATGGC<br>Reverse: CGGTTCTGTGGATCTAGC	hypothalamus
Neuropeptide Y receptor Y2	***Npy2r***	Forward: CATCTTCACCGTGTTCCACA<br>Reverse: AAGGCCGAGAGGAAAGCTTT	hypothalamus
Growth hormone secretagogue receptor	***Ghsr***	Forward: CTCAGGGACCAGAACCACAAAC<br>Reverse: ACAAAGGACACCAGGTTGCAG	hypothalamus
Lysozyme 1	***Lyz1***	Forward: GCCAAGGTCTACAATCGTTGTGAGTT<br>Reverse: CAGTCAGCCAGCTTGACACCACG	illeum and colon
Defensin A	***DefA***	Forward: GGTGATCATCAGACCCCAGCATCAGT<br>Reverse: AAGAGACTAAAACTGAGGAGCAGC	ileum and colon
Regenerating islet-derived protein 3 gamma	***Reg3g***	Forward: TTCCTGTCCTCCATGATCAAA<br>Reverse: CATCCACCTCTGTTGGGTTC	ileum and colon
Marker of proliferation Ki-67	***Ki67***	Forward: CAGACTTGCTCTGGCCTACC<br>Reverse: GGTTGGCGTTTCTCCTCTTT	ileum and colon
Occludin	***Ocln***	Forward: ATGTCCGGCCGATGCTCTC<br>Reverse: TTTGGCTGCTCTTGGGTCTGTAT	ileum and colon
Claudin 3	***Cldn3***	Forward: TCATCGGCAGCAGCATCATCAC<br>Reverse: ACGATGGTGATCTTGGCCTTGG	ileum and colon
Adrenergic receptor, beta 2	***Adrb2***	Forward: CAAGTTCGAGCGACTACAAA<br>Reverse: CTTGAAGGGCGATGTGATAG	white adipose tissue (epididymal)
Adrenergic receptor, beta 2	***Adrb3***	Forward: TAGGGAAAAGAGAGCACCCC<br>Reverse: TGGTCCAAGATGGTGCTTAG	white adipose tissue (epididymal)
Ribosomal protein L19	***Rpl19***	Forward: CCTTGTCTGCCTTCAGCTTGT<br>Reverse: GAAGGTCAAAGGGAATGTGTTCA	housekeeping

Conditions of the qPCR reactions - denaturation: 95°C (10 min); 45 amplification cycles: 95°C (10 s), 60°C (30 s), and 72°C (5 s); final melting stage: 95°C (5 s), and 65°C (1 min)
All primer pairs were supplied by Isogen Life Science except primers for *Glp1r* amplification, which were supplied by Sigma.

Supplementary Figures

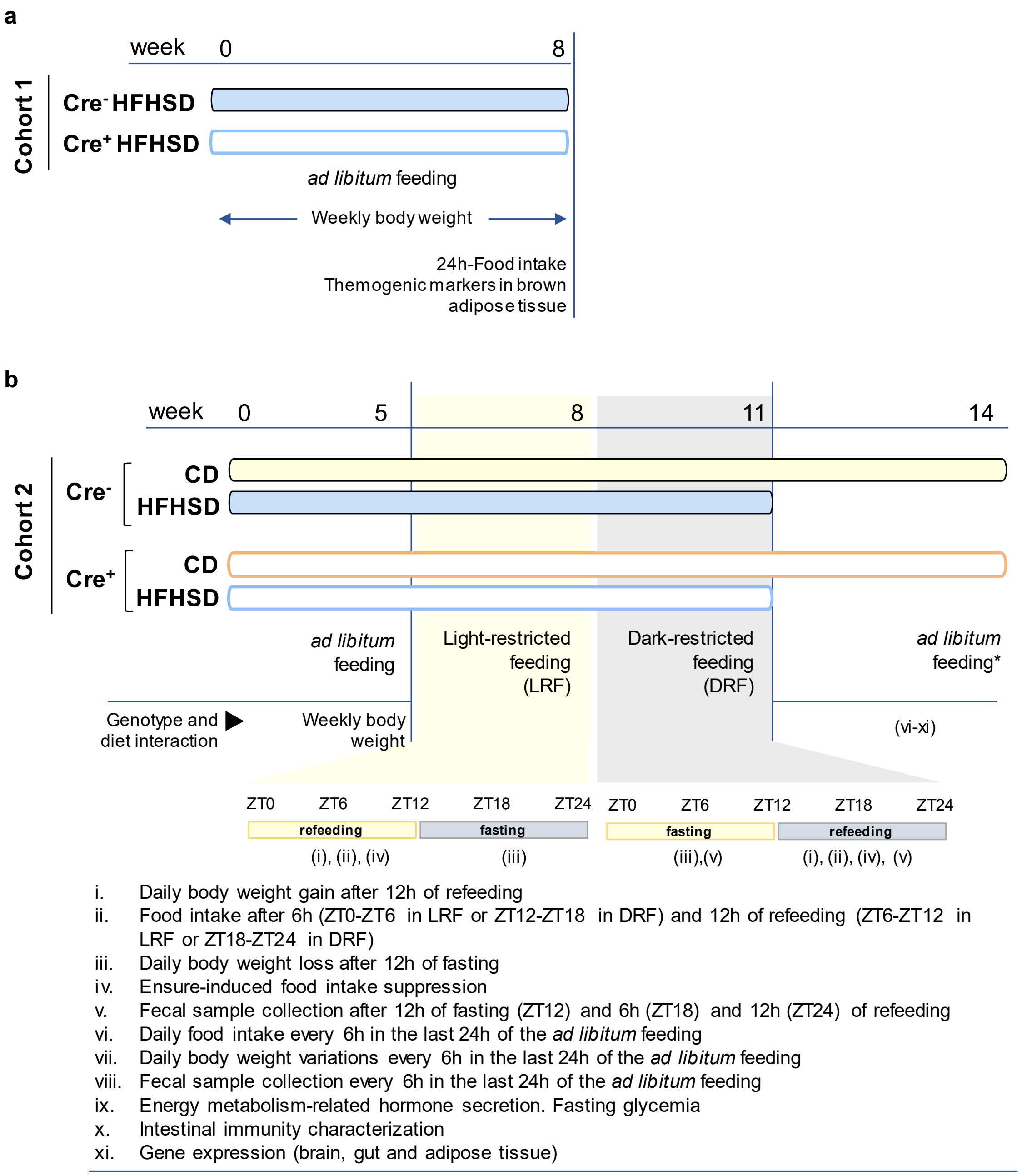

Supplementary Figure 1: Graphical representation of the experimental procedure
a In cohort 1, mice lacking Nav1.8+ neurons (Cre$^+$) and their control littermates (Cre$^-$) were fed a high-fat high-sugar diet (HFHSD) for 8 weeks (Cre$^-$ HFHSD: n = 6 and Cre$^+$ HFHSD: n = 4 mice). **b** In cohort 2, Cre$^+$ and Cre$^-$ mice were randomly allocated to control diet (CD) or HFHSD groups (Cre$^-$ CD/HFHSD: n = 10 per group; and Cre+ CD/HFHSD: n = 6 and n = 7 mice, respectively). Mice were submitted to different feeding schedules along 14 weeks: *ad libitum* feeding (5 weeks), followed by light- and dark-restricted feeding (LRF/DRF) (3 weeks each) and *ad libitum* feeding (3 weeks). Roman numerals indicate functional assessments and molecular/cellular analyses performed at each

feeding schedule at specified zeitgeber time (ZT), with ZT0-ZT12 and ZT12-ZT24 corresponding to the ZTs of the light and dark phases, respectively. Measurements of the last *ad libitum* feeding are only shown for CD-fed mice (Cre$^-$/Cre$^+$) as HFHSD-*ad libitum* feeding profoundly reduced the survival of Cre$^+$ mice.

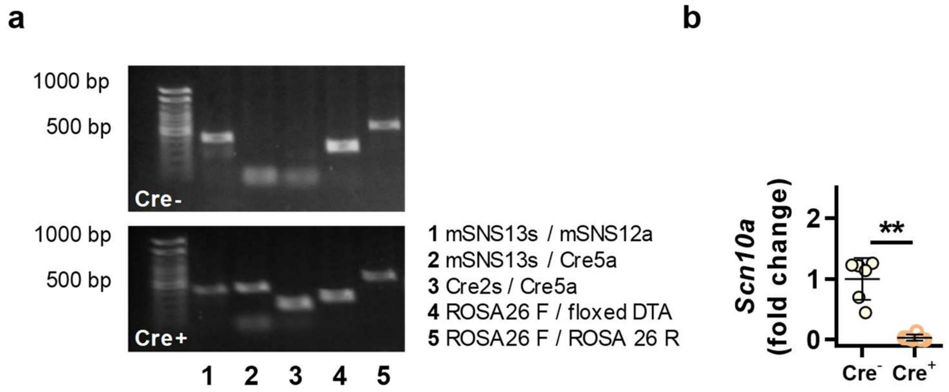

Supplementary Figure 2: Validation of the ablation of Nav1.8+ cells in the offspring by diphtheria toxin A (DTA)-targeted expression based on the Cre-LoxP system

a Electrophoretic separation of amplified DNA fragments (base pairs, bp) of DTA and Cre recombinase (presented in approximately half of the mice). **b** Silenced gene expression of *Nav1.8* in nodose ganglia of Cre$^+$ mice (Nav1.8-cre/DTA) relative to that observed in control littermates (Cre$^-$). Results are represented as scatter plots indicating individual values with mean ± SEM (n = 5 mice). Mice fed CD are depicted by solid yellow (Cre$^-$) or empty orange (Cre$^-$) circles.

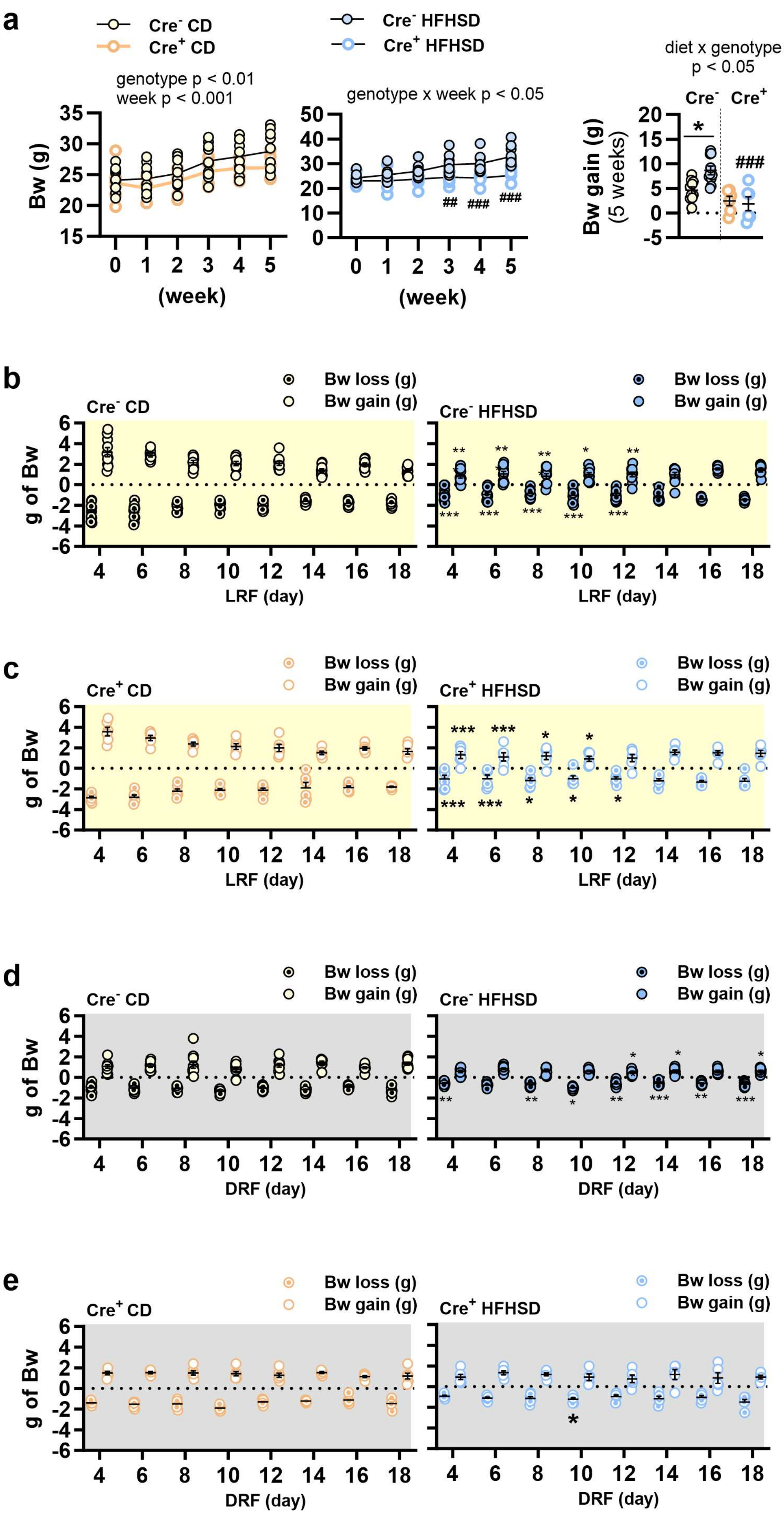
a
Cre- CD
Cre+ CD
Cre- HFHSD
Cre+ HFHSD
genotype p < 0.01
week p < 0.001
genotype x week p < 0.05
diet x genotype
p < 0.05
Bw (g)
(week)
###
Cre- Cre+
*
###
Bw gain (g)
(5 weeks)
b
Cre- CD
Cre- HFHSD
Bw loss (g)
Bw gain (g)
g of Bw
LRF (day)
c
Cre+ CD
Cre+ HFHSD
Bw loss (g)
Bw gain (g)
g of Bw
LRF (day)
d
Cre- CD
Cre- HFHSD
Bw loss (g)
Bw gain (g)
g of Bw
DRF (day)
e
Cre+ CD
Cre+ HFHSD
Bw loss (g)
Bw gain (g)
g of Bw
DRF (day)

Supplementary Figure 3 (related to Figure 2)

a Body weight (Bw) follow-up and Bw gain of control mice (Cre$^-$) and Nav1.8-cre/DTA mice (Cre$^+$) fed *ad libitum* with either control diet (CD) or high fat high sugar diet (HFHSD) for 5 weeks.

12h-body weight gain (positive values) and body weight loss (negative values) every 2 days in:

b Control mice fed CD or HFHSD during the light restricted feeding (LRF). **c** Nav1.8-cre/DTA mice fed CD or HFHSD during LRF. **d** Control mice fed CD or HFHSD during DRF. **e** Nav1.8-cre/DTA mice fed CD or HFHSD along DRF. Data are represented as scatter plots for individual values with mean follow-up curve or as scatter plots indicating individual values ± SEM (Cre$^-$ CD and Cre$^-$ HFHSD, n = 10 mice; Cre$^+$ CD, n = 6 mice; Cre$^+$ HFHSD, n = 5-7 mice. Mice fed CD are depicted by solid yellow (Cre$^-$) or empty orange (Cre$^-$) circles, mice fed HFHSD are depicted by solid blue (Cre$^-$) or empty blue (Cre$^-$) circles; shadings in light yellow and grey represent LRF and DRF regimes, respectively. (**a**): Two-way ANOVA with genotype (Cre$^-$ or Cre$^+$) and diet (CD/HFHSD) as between-subject factor; (**b-e**): Two-way ANOVA with diet (CD or HFHSD) and day of the LRF or DRF as between-subject factors. Bonferroni's *post hoc* test. a ## p < 0.01 and ### p < 0.001 vs Cre$^-$ HFHSD and * p < 0.05 vs Cre$^-$ CD; (b-e) * p < 0.05, ** p < 0.01 and *** p < 0.001 vs either Cre$^-$ CD or Cre$^+$ CD

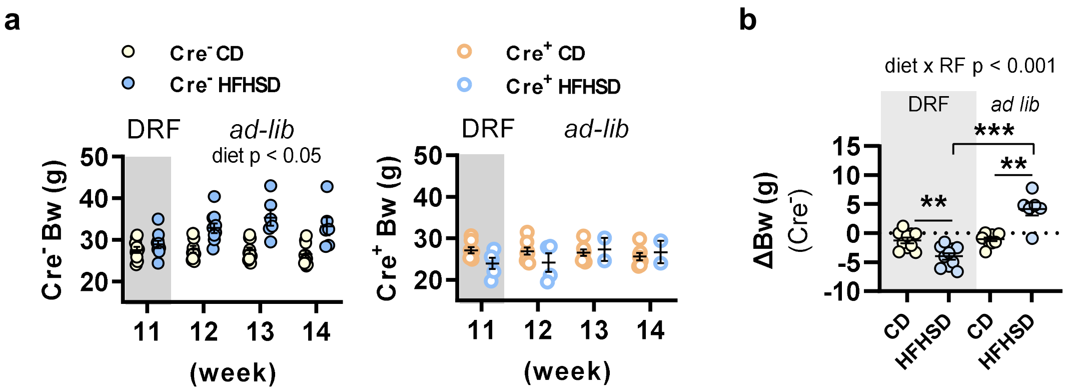

Supplementary Figure 4: Body weight changes under 24-hour free access to food conditions during 3 weeks

a Body weight (Bw) follow-up along the last 3 weeks of the experiment when control mice (Cre$^-$) and mice lacking Nav1.8+ neurons (Cre$^+$) on either control diet (CD) or high-fat high-sugar diet (HFHSD) were switched from dark-restricted feeding (DRF) to *ad libitum* feeding (*ad-lib*). **b** Impact of the *ad libitum* feeding reverting the effects of DRF on Bw:

calculated Bw differences from the end of the *ad-lib* feeding and the DRF (ΔBw). Results are represented as scatter plots indicating individual values with mean ± SEM. (Cre$^-$ CD and Cre$^-$HFHSD, n = 5–10 mice; Cre$^+$ CD, n = 5–6 mice; Cre$^+$ HFHSD, n = 2-5 mice). Mice fed CD are depicted by solid yellow (Cre$^-$) or empty orange (Cre$^-$) circles, mice fed HFHSD are depicted by solid blue (Cre$^-$) or empty blue (Cre$^-$) circles; shading in grey represents DRF regime. Two-way ANOVA with genotype (Cre$^-$ or Cre$^+$) and diet (CD or HFHSD) as between-subject factors followed by Bonferroni's *post hoc* test.

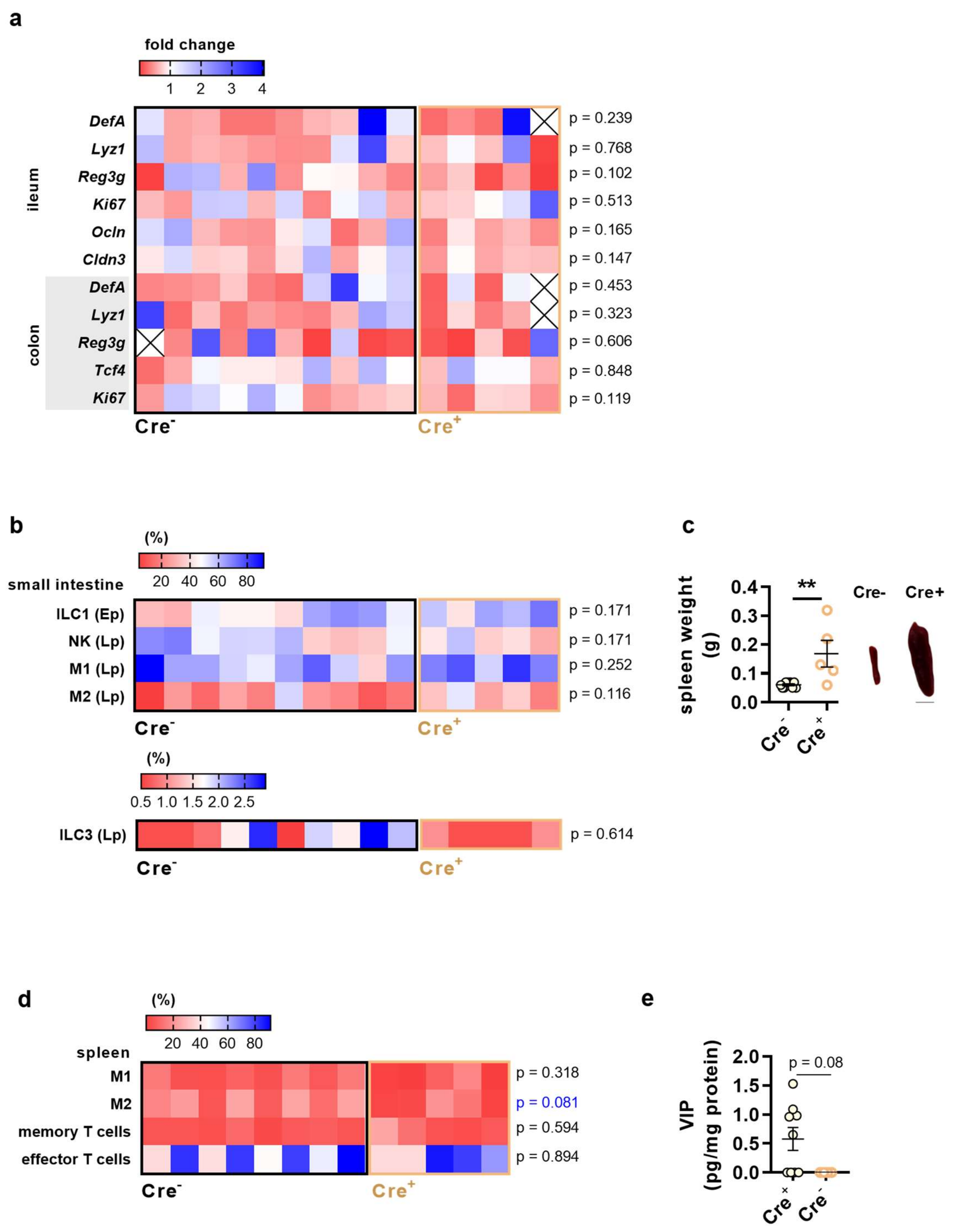

Supplementary Figure 5 (related to Figure 5) **Analysis of immune markers at the end of the experiments in mice fed control diet**

a Gene expression of antimicrobial peptides (*DefA*, *Lyz1*, and *Reg3g*), tight junction proteins (*Ocln* and *Cldn3*) and markers of proliferation (*Ki67*) and differentiation (*Tcf4*) in ileum and colon, represented as fold-change of the gene expression relative to control littermates. **b** Percentage of type 1 innate lymphoid cells (ILC1), natural killer cells (NK), and type 1 and type 2 macrophages (M1 and M2, respectively) in the lamina propria of

the small intestine. **c** Weight of the spleen, scale bar: 0.5 cm. **d** Percentage of M1, M2, and memory and effector T cells in the spleen. **e** Vasoactive intestinal peptide (VIP) concentration in ileum. Results are shown in a heatmap in which color intensity represents the relative gene expression (**a**) or the relative abundance of immune cells expressed as a percentage (**b**, **d**), and missing values are indicated as “X” (**a**, **b**, **d**); or shown as scatter plots indicating individual values with mean ± SEM (**c**, **e**) (Cre^- n = 9–10 mice and Cre^+ n = 5 mice). Mice fed CD are depicted by solid yellow (Cre^-) or empty orange (Cre^-) circles. All measurements were analyzed using Student’s t test except for (**c**), where mRNA levels of *DefA*, *Lyz1* and *Ki67* in ileum and *DefA* and *Reg3g* in colon were analyzed with the Mann-Whitney U test. **p < 0.01.

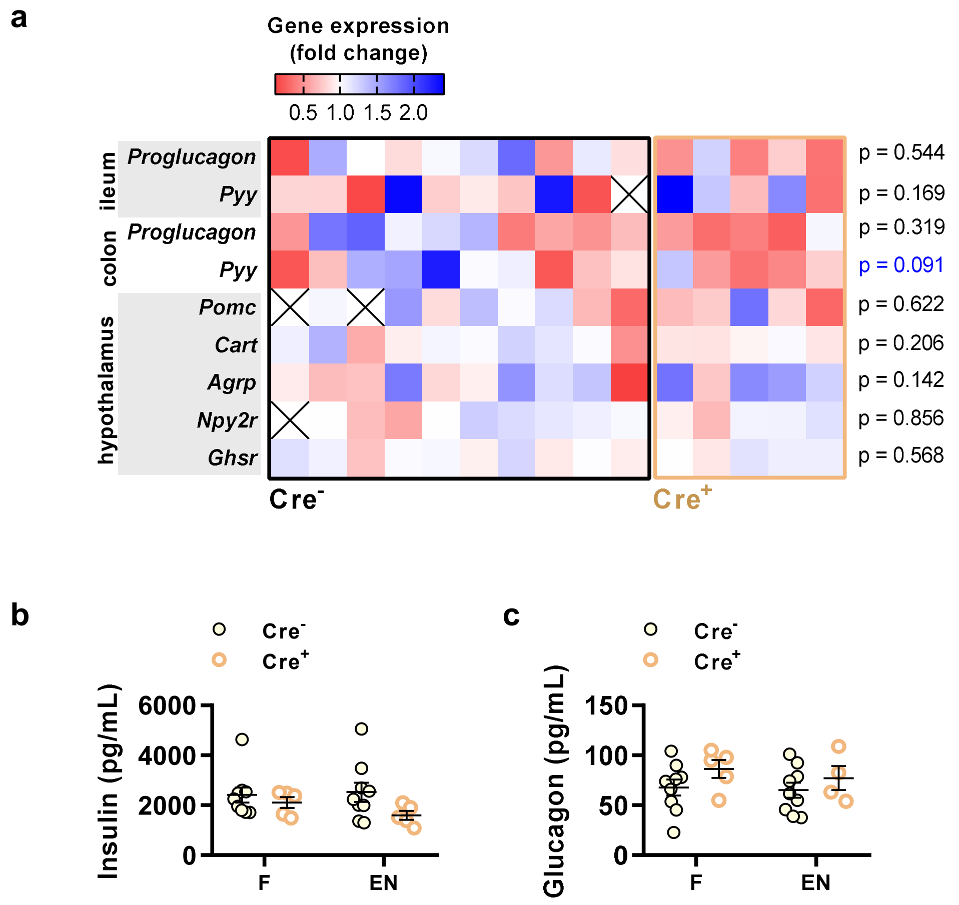

Supplementary Figure 6 (related to Figure 6 and 7). **Gene expression of hypothalamic markers involved in food intake control and plasma levels of hormones involved in regulating glycemia**

In mice lacking Nav1.8+ neurons (Cre^+) and their control littermates (Cre^-) fed control diet (CD) we analyzed at the end of the experiment: **a** mRNA levels of different genes including the precursor of GLP-1 (*Proglucagon*) and peptide tyrosine tyrosine (*Pyy*) in ileum and colon, and food intake-related peptides and receptors in the hypothalamus

(including *Pomc*, *Cart*, *Agrp*, *Npy2r*, and *Ghsr*). The heatmap shows the gene expression variations, represented by fold-change relative to control littermates; color intensity depicts the relative gene expression, and missing values are indicated as “X”. **b**, **c** Plasma insulin and glucagon levels in 4-h fasted f mice and 15 min after an oral gavage of the nutrient mixed solution (ensure, EN). Results are represented as scatter plots indicating individual values with mean ± SEM. (Cre^- n = 6–10 mice and Cre^+ n = 5 mice). Mice fed CD are depicted by solid yellow (Cre^-) or empty orange (Cre^-) circles. (**a**) Student´s t test. (**b**, **c**): Two-way ANOVA with genotype (Cre^- or Cre^+) and prandial condition (fasting/ensure, F/EN) as between-subject factor.

a ILC1 group (intestinal epithelium)

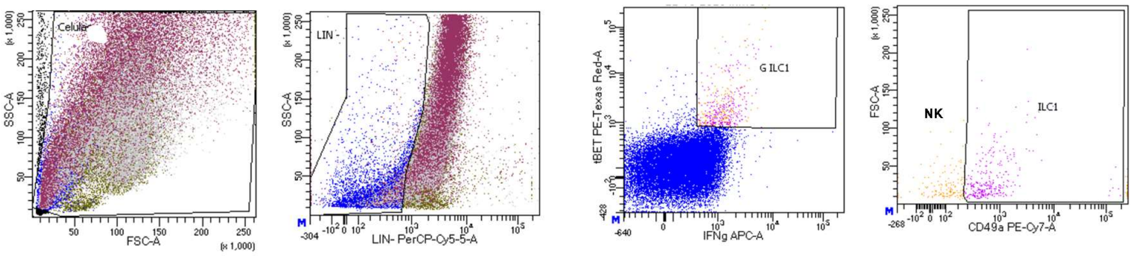

b ILC2 and ILC3 group (intestinal lamina propria)

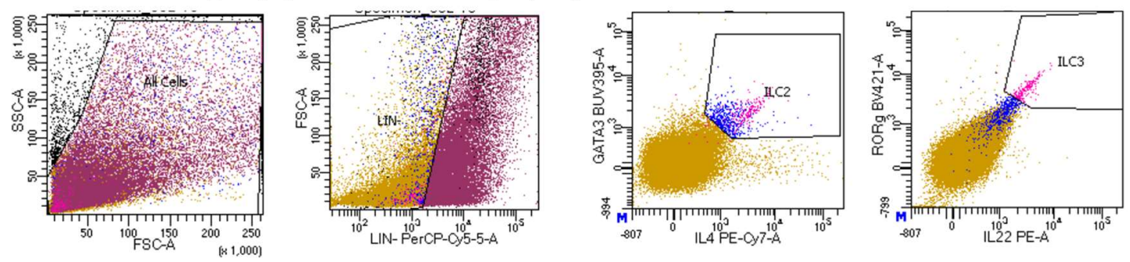

c Macrophages (intestinal lamina propria)

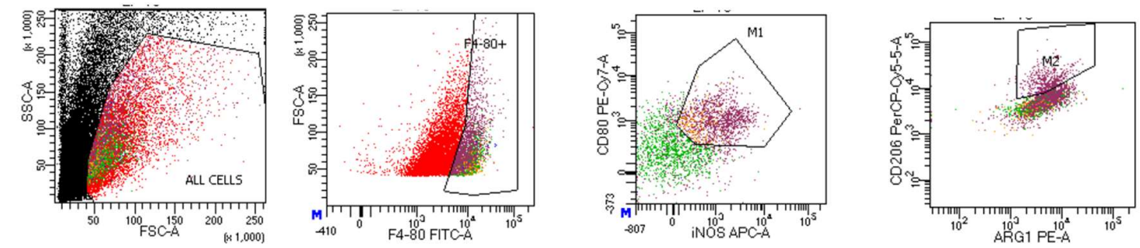

d Treg and Th17 (intestinal lamina propria)

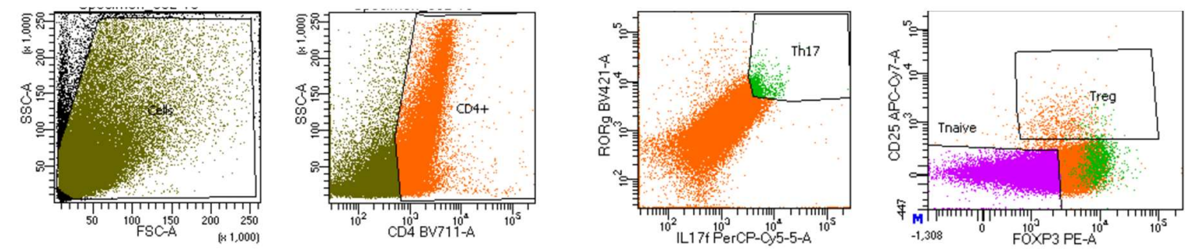

e Macrophages (spleen)

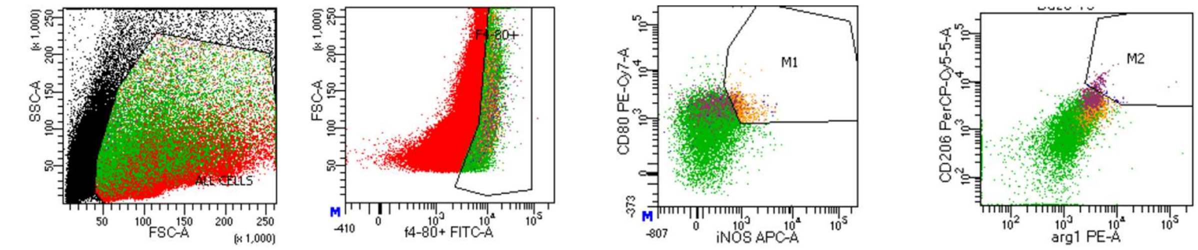

f Memory and effector T cells (spleen)

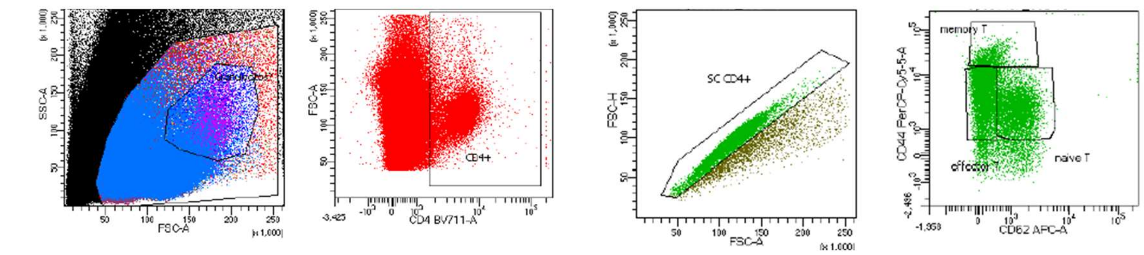

Supplementary Figure 7: Gating strategy for flow cytometry analysis of immune cells in mice fed control diet

a Gating strategy for type 1 innate lymphoid cells (ILC1) and natural killer cells (NK) in the intestinal epithelium. Total cells were selected from a FSC-A vs SSC-A dot plot, and lineage negative $(LIN)^-$ cells were selected using a mouse lineage antibody cocktail. Subsequently, ILC1 group was characterized as $Tbet^+$ $IFN\gamma^+$, which was subsequently selected for CD49a expression as ILC1, while $CD49a^-$ cells were characterized as NK. **b** Gating strategy for type 2 and type 3 innate lymphoid cells (ILC2 and ILC3, respectively) in the intestinal lamina propria. LIN^- cells were selected as for ILC1 group (**a**). ILC2 were then characterized as $GATA3^+$ $IL4^+$, and ILC3 were identified as $ROR\gamma t^+$ $IL22^+$. **c** Gating strategy for macrophages in the intestinal lamina propria. $F4/80^+$ cells were selected from FSC-A vs SSC-A dot plot. M1 and M2 macrophages were the characterized as $iNOS^+CD80^+$ and $Arg1^+CD206^+$, respectively. **d** Gating strategy for Treg and Th17 in the intestinal lamina propria. $CD4^+$ cells were selected from FSC-A vs SSC-A dot plot. $CD4^+$ cells were selected to subsequently identify $IL17F^+$ $ROR\gamma t^+$ population (Th17) and $CD25^+$ $FoxP3^+$ cells (Treg). **e** Gating strategy for macrophages in the spleen. $F4/80^+$ cells were selected from FSC-A vs SSC-A dot plot. M1 and M2 macrophages were the characterized as $iNOS^+CD80^+$ and $Arg1^+$ $CD206^+$, respectively. **f** Gating strategy for memory and effector T cells in the spleen. $CD4^+$ cells were selected from a FSC-A vs SSC-A dot plot. $CD4^+$ cells were selected to subsequently identify CD44highCD62low as memory T cells and $CD44^-CD62^-$ as effector T cells.
