# Supplementary material for: Nav1.8-expressing neurons control daily oscillations of food intake, body weight and gut microbiota in mice
Source: Commun Biol. 2024 Feb 22;7:219. doi: 10.1038/s42003-024-05905-3 (PMC10883928; doi:10.1038/s42003-024-05905-3)
Supplement: Supplementary file 3 — Description of Additional Supplementary Files [file 42003_2024_5905_MOESM3_ESM.pdf]

Description of Additional Supplementary Files

File name: Supplementary Data 1
Description: The source of data for the graphs and charts in the paper.
