# Supplementary material for: Nav1.8-expressing neurons control daily oscillations of food intake, body weight and gut microbiota in mice
Source: Commun Biol. 2024 Feb 22;7:219. doi: 10.1038/s42003-024-05905-3 (PMC10883928; doi:10.1038/s42003-024-05905-3)
Supplement: Supplementary file 5 — Reporting Summary [file 42003_2024_5905_MOESM5_ESM.pdf]

nature portfolio

Corresponding author(s): MRP

Last updated by author(s): Jan 30, 2024

Reporting Summary

Nature Portfolio wishes to improve the reproducibility of the work that we publish. This form provides structure for consistency and transparency in reporting. For further information on Nature Portfolio policies, see our Editorial Policies and the Editorial Policy Checklist.

Statistics

For all statistical analyses, confirm that the following items are present in the figure legend, table legend, main text, or Methods section.

n/a	Confirmed	
☐	☒	The exact sample size (*n*) for each experimental group/condition, given as a discrete number and unit of measurement
☐	☒	A statement on whether measurements were taken from distinct samples or whether the same sample was measured repeatedly
☐	☒	The statistical test(s) used AND whether they are one- or two-sided<br>*Only common tests should be described solely by name; describe more complex techniques in the Methods section.*
☐	☒	A description of all covariates tested
☐	☒	A description of any assumptions or corrections, such as tests of normality and adjustment for multiple comparisons
☐	☒	A full description of the statistical parameters including central tendency (e.g. means) or other basic estimates (e.g. regression coefficient) AND variation (e.g. standard deviation) or associated estimates of uncertainty (e.g. confidence intervals)
☐	☒	For null hypothesis testing, the test statistic (e.g. *F*, *t*, *r*) with confidence intervals, effect sizes, degrees of freedom and *P* value noted<br>*Give P values as exact values whenever suitable.*
☒	☐	For Bayesian analysis, information on the choice of priors and Markov chain Monte Carlo settings
☐	☒	For hierarchical and complex designs, identification of the appropriate level for tests and full reporting of outcomes
☐	☒	Estimates of effect sizes (e.g. Cohen's *d*, Pearson's *r*), indicating how they were calculated

Our web collection on statistics for biologists contains articles on many of the points above.

Software and code

Policy information about availability of computer code

Data collection	not software was used
Data analysis	GraphPad Software, Inc. (9.0.0), edgeR v.1.38 R package, DiscoRhythm

For manuscripts utilizing custom algorithms or software that are central to the research but not yet described in published literature, software must be made available to editors and reviewers. We strongly encourage code deposition in a community repository (e.g. GitHub). See the Nature Portfolio guidelines for submitting code & software for further information.

Data

Policy information about availability of data

All manuscripts must include a data availability statement. This statement should provide the following information, where applicable:

- Accession codes, unique identifiers, or web links for publicly available datasets
- A description of any restrictions on data availability
- For clinical datasets or third party data, please ensure that the statement adheres to our policy

Raw data from 16S rRNA amplicon sequencing has been deposited at European Nucleotide Archive (ENA). Accession number: PRJEB58911. The procedures for obtaining source data can be found in the general guide on ENA data retrieval. Source data for the graphs and charts are provided as Supplementary Data 1. Any remaining information can be obtained from the corresponding author upon reasonable request.

Research involving human participants, their data, or biological material

Policy information about studies with human participants or human data. See also policy information about sex, gender (identity/presentation), and sexual orientation and race, ethnicity and racism.

Reporting on sex and gender	this study did not involve human research participants
Reporting on race, ethnicity, or other socially relevant groupings	this study did not involve human research participants
Population characteristics	this study did not involve human research participants
Recruitment	this study did not involve human research participants
Ethics oversight	this study did not involve human research participants

Note that full information on the approval of the study protocol must also be provided in the manuscript.

Field-specific reporting

Please select the one below that is the best fit for your research. If you are not sure, read the appropriate sections before making your selection.

☒ Life sciences ☐ Behavioural & social sciences ☐ Ecological, evolutionary & environmental sciences

For a reference copy of the document with all sections, see nature.com/documents/nr-reporting-summary-flat.pdf

Life sciences study design

All studies must disclose on these points even when the disclosure is negative.

Sample size	Sample size was calculated using the G*Power software based on a previously estimated effect size (Romaní-pérez M et al FASEB J. 2021 Jul;35(7):e21734. doi: 10.1096/fj.202100126R)
Data exclusions	Outliers, identified using GraphPad Software, Inc. (9.0.0), were excluded from the analyses.
Replication	eplicated measures of all variables verified the reproducibility of the experimental findings: Fod intake and body weight variations were measured in two consecutive days. Glycemia, plasma hormone levels, gene expression (RNA levels) in tissues were measured in duplicate.
Randomization	Mice, with similar body weight, were randomly allocated into the experimental groups by the animal health care technicians
Blinding	Allocation, data collection and analyses were conducted under blinding conditions

Reporting for specific materials, systems and methods

We require information from authors about some types of materials, experimental systems and methods used in many studies. Here, indicate whether each material, system or method listed is relevant to your study. If you are not sure if a list item applies to your research, read the appropriate section before selecting a response.

Materials & experimental systems

n/a	Involved in the study	
☐	☒	Antibodies
☒	☐	Eukaryotic cell lines
☒	☐	Palaeontology and archaeology
☐	☒	Animals and other organisms
☒	☐	Clinical data
☒	☐	Dual use research of concern
☒	☐	Plants

Methods

n/a	Involved in the study	
☒	☐	ChIP-seq
☐	☒	Flow cytometry
☒	☐	MRI-based neuroimaging

Antibodies

Antibodies used	PerCP-Cy™5.5-conjugated anti-Lineage antibody cocktail (LIN), PE-Vio615-conjugated anti- Tbet, APC-conjugated anti-IFNɤ, PE-vio 770- conjugated anti-CD49a, BUV395- conjugated anti-GATA3, Pe-Cy7- conjugated anti-IL4, BV421- conjugated anti- RORɤt, PE-conjugated anti-IL22, FITC-conjugated anti-F4/80+, Pe-Vio770-conjugated anti-CD80, APC-conjugated anti-iNOS, PerCPCy5.5-

	conjugated anti-CD206, PE-conjugated anti-Arg1, BV711-conjugated anti-CD4, APCCy7-conjugated anti-CD25, PE-conjugated anti-Foxp3, PerCP-Cy5.5 conjugated anti-IL17F, PerCP-Cy™5.5-conjugated anti-CD44, APC-conjugated anti-CD62
Validation	Antibodies for flow cytometry were already validated in previous studies (Romaní-pérez M et al FASEB J. 2021 Jul;35(7):e21734. doi: 10.1096/fj.202100126R and López-Almela et al Gut Microbes. 2021 Jan-Dec;13(1):1-20. doi: 10.1080/19490976.2020.1865706)

Animals and other research organisms

Policy information about studies involving animals; ARRIVE guidelines recommended for reporting animal research, and Sex and Gender in Research

Laboratory animals	This work used transgenic mice of 6 weeks old obtained by crossing heterozygous Nav1.8 knock-in Cre-recombinase male mice (EM:04582, EMMA/Infrafrontier repository, Munich, Germany) with homozygous ROSA26-eGFP-DTA female mice (stock #006331; The Jackson Laboratory, Bar Harbor, ME) with C57BL/6J genetic background
Wild animals	This study did not involved the use of wild animals
Reporting on sex	The sex of the offspring mice was assigned by presence or absence of testicles after weaning. This study only includes males since females were used for another study.
Field-collected samples	This study did not involve samples collected from the field
Ethics oversight	All experimental procedures using animals were in accordance with European Union 2010/63/UE and Spanish RD53/2013 guidelines and approved by the ethics committee of the University of Valencia (Animal Production Section, SCSIE, University of Valencia, Spain) and authorized by Dirección General de Agricultura, Ganadería y Pesca (Generalitat Valenciana) (approval ID 2019/VSC/PEA/ 0020 and 2020/VSC/PEA/0022)

Note that full information on the approval of the study protocol must also be provided in the manuscript.

Plants

Seed stocks	this study did not involve plants
Novel plant genotypes	this study did not involve plants
Authentication	this study did not involve plants

Flow Cytometry

Plots

Confirm that:

- ☒ The axis labels state the marker and fluorochrome used (e.g. CD4-FITC).
- ☒ The axis scales are clearly visible. Include numbers along axes only for bottom left plot of group (a 'group' is an analysis of identical markers).
- ☒ All plots are contour plots with outliers or pseudocolor plots.
- ☒ A numerical value for number of cells or percentage (with statistics) is provided.

Methodology

Sample preparation	The procedure to isolate immune cells from the intestinal epithelium and the lamina propria is detailed in methods section
Instrument	BD LSRFortessa flow cytometer operated with (BD Biosciences)
Software	FACS Diva software v.7.0 (BD Biosciences)
Cell population abundance	The abundance of the relevant cell populations was represented as the parent percentage (% parent is defined as the number of gated events divided by the number of events for the parent). For the populations described in this work, we have used the following parent population: Lin- for ILCs, F4/80 for M1 and M2 macrophages and CD4+ for Treg, Th17, Memory T cells and Effector T cells. These markers were combined with specific markers as described in Supplementary Table 2.<br>The purity of the samples was determined using the unlabeled study samples and a labeled control group. See Table S2 in

supplemental information

Gating strategy

Previously to the selection of parent cell populations discussed in the previous section, we carried out the following gating strategy. The first step in gating was distinguishing populations of cells based on their forward (FSC) and side scatter (SSC) properties. The next step was to discriminate when two or more cells were stuck together as they passed through the flow cell. We used FSC-A x FSC-H to eliminate “doublets”. Finally, we select living cells using a cell viability marker

☒ Tick this box to confirm that a figure exemplifying the gating strategy is provided in the Supplementary Information.
